# Rapamycin maintains NAD^+^/NADH redox homeostasis in muscle cells

**DOI:** 10.18632/aging.103954

**Published:** 2020-09-22

**Authors:** Zhigang Zhang, He N. Xu, Siyu Li, Antonio Davila Jr, Karthikeyani Chellappa, James G. Davis, Yuxia Guan, David W. Frederick, Weiqing Chu, Huaqing Zhao, Lin Z. Li, Joseph A. Baur

**Affiliations:** 1 College of Veterinary Medicine, Northeast Agricultural University, Harbin 150030, China; 2 Institute for Diabetes, Obesity, and Metabolism, Department of Physiology, Perelman School of Medicine, University of Pennsylvania, Philadelphia, PA 19104, USA; 3 Britton Chance Laboratory of Redox Imaging, Department of Radiology, Perelman School of Medicine, University of Pennsylvania, Philadelphia, PA 19104, USA; 4 Division of Trauma, Critical Care, and Emergency Surgery, University of Pennsylvania, Philadelphia, PA 19104, USA; 5 Department of Clinical Sciences, Temple University School of Medicine, Philadelphia, PA 19104, USA; 6 Institute of Translational Medicine and Therapeutics, University of Pennsylvania, Philadelphia, PA 19104, USA

**Keywords:** rapamycin, optical redox imaging, aging, NAD^+^/NADH ratio, redox state

## Abstract

Rapamycin delays multiple age-related conditions and extends lifespan in organisms ranging from yeast to mice. However, the mechanisms by which rapamycin influences longevity are incompletely understood. The objective of this study was to investigate the effect of rapamycin on NAD^+^/NADH redox balance. We report that the NAD^+^/NADH ratio of C2C12 myoblasts or differentiated myotubes significantly decreases over time in culture, and that rapamycin prevents this effect. Despite lowering the NADH available to support ATP generation, rapamycin increases ATP availability, consistent with lowering energetic demand. Although rapamycin did not change the NAD^+^/NADH ratio or steady-state ATP concentration in the livers, kidneys, or muscles of young mice, optical redox imaging revealed that rapamycin caused a substantial decline in the NADH content and an increase in the optical redox ratio (a surrogate of NAD^+^/NADH redox ratio) in muscles from aged mice. Collectively, these data suggest that rapamycin favors a more oxidized NAD^+^/NADH ratio in aged muscle, which may influence metabolism and the activity of NAD^+^-dependent enzymes. This study provides new insight into the mechanisms by which rapamycin might influence the aging process to improve health and longevity among the aging population.

## INTRODUCTION

It is estimated that the fraction of the global population over the age of 60 years will reach 20% in the near future, and health-care costs will rise correspondingly [1]. Thus, there is a growing recognition that solutions must be found to keep people healthy longer. Aging is a complex and multifaceted process. Nevertheless, research has demonstrated that health and longevity can be extended by calorie restriction [2], medications such as metformin [3], rapamycin [4], ibuprofen [5], resveratrol [6], spermidine [7], and supplementation of nicotinamide adenine dinucleotide (NAD^+^) [8], exposure to young blood [9], transfer of extracellular vesicles containing nicotinamide phosphoribosyltransferase [10]*,* or elimination of senescent cells [11]. More recently, Fahy et al. reported that epigenetic aging can be reversed in human cells *in vivo* using a cocktail of drugs that was designed to promote regeneration of the thymus [12]. Collectively, these observations raise hopes that intervention in human aging may be possible.

Rapamycin and its analogs are clinically approved drugs that prevent solid organ allograft rejection and are used in the treatment of certain cancers. Rapamycin is an inhibitor of mechanistic target of rapamycin (mTOR), which has wide-ranging effects on growth and metabolism across tissues. Rapamycin extends maximal lifespan in model organisms ranging from yeast to mice, and a few studies suggest that rapamycin may also promote healthy aging in humans [4, 13, 14]*.* In addition to promoting survival, rapamycin is also protective in several mouse models of chronic disease [1, 15], and suppresses geroconversion which is a conversion from reversible cell cycle arrest to irreversible senescence [16]. Despite extensive studies, it remains unclear how exactly rapamycin affects longevity and diseases.

Maintaining NAD^+^ redox balance is necessary for optimum cellular health and may be compromised over the course of natural aging. NAD^+^, which is interconverted between its oxidized (NAD^+^) and reduced (NADH) forms, is an endogenous coenzyme and co-substrate that has key roles in diverse cellular and physiologic processes including energy metabolism and signaling through enzymes such as poly ADP-ribose polymerases (PARPs) and sirtuins. The NAD^+^/NADH ratio in a given cellular compartment represents the redox state, which is influenced by, and in turn regulates, metabolic activity [12]. A decrease in the NAD^+^/NADH ratio, reduced cellular NAD^+^ level, and increased NADH have all been observed during aging [17–19]. However, the effect of rapamycin on the NAD^+^/NADH redox state has not been thoroughly investigated.

The cell has two major routes to re-oxidize NADH to NAD^+^, the mitochondrial electron transport chain and lactate dehydrogenase (with subsequent export of lactate). In the absence of a functional electron transport chain, cultured cells are completely dependent on a source of exogenous oxidizing equivalents (typically pyruvate) to oxidize NADH and maintain growth [20, 21]. Under conditions of high cell density due to long-term culture or high seeding density, the release of lactic acid produced via aerobic glycolysis, which is necessary to dispose of excess reducing equivalents, acidifies the culture media. High lactate and low extracellular pH may feed back to inhibit the lactate dehydrogenase reaction, preventing cells from regenerating NAD^+^ from NADH and thus resulting in a reduced redox ratio, similar to that observed in aged tissues *in vivo* [18, 22–24]. Interestingly, rapamycin has been shown to decrease lactate production in cultured cells, raising the possibility that it can also shift NAD redox balance [25].

In the present study, we employ multiple methods, including the optical redox imaging techniques pioneered by Chance et al. [26–29], to investigate the effects of rapamycin on NAD^+^/NADH redox status in cultured cells, and in old and young mice in order to gain a more complete understanding of the mechanisms by which rapamycin may influence mammalian physiology.

## RESULTS

### NAD^+^/NADH ratios were decreased in C2C12 myoblasts cultured at high density

**Figure 1:**
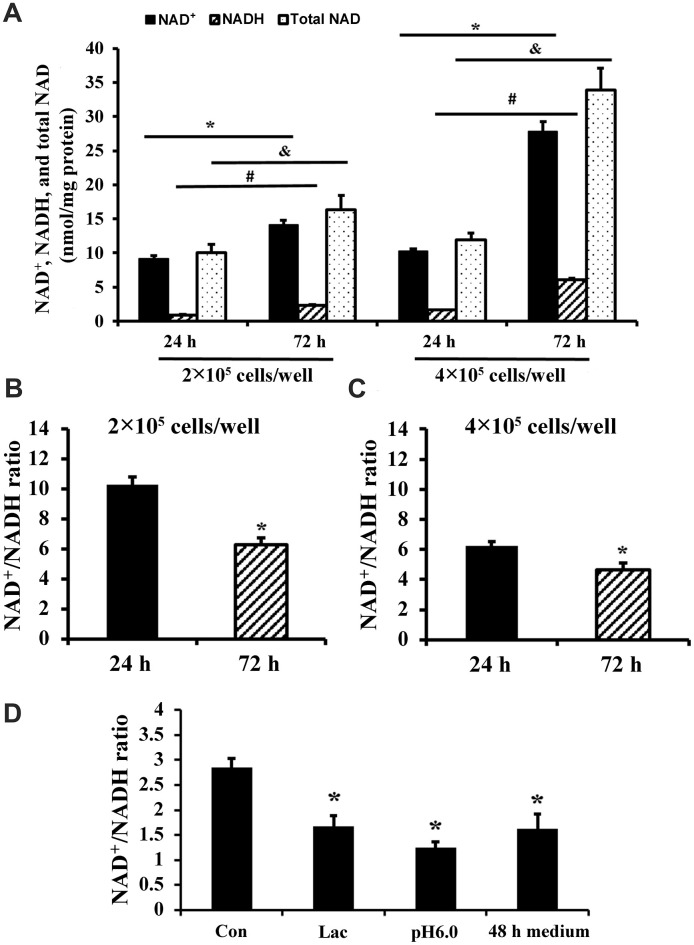
**NAD^+^/NADH ratios in long-term cultured C2C12 myoblasts.** (**A**) NAD^+^, NADH, and total NAD concentrations of C2C12 myoblasts (2×10^5^ cells/well and 4×10^5^ cells/well) cultured for 24 and 72 h, respectively. *, *P* < 0.05 for comparison of NAD^+^ concentrations between cells cultured for 24 h and 72 h; #, *P* < 0.05 for comparison of NADH concentrations between cells cultured for 24 h and 72 h. &, *P* < 0.05 for comparison of total NAD^+^ concentrations between cells cultured for 24 h and 72 h. (**B**) NAD^+^/NADH ratio of C2C12 myoblasts (2×10^5^ cells/well) cultured for 24 and 72 h; *, *P* < 0.05 versus cells cultured for 24 h group. (**C**) NAD^+^/NADH ratio of C2C12 myoblasts (4×10^5^ cells/well) cultured for 24 and 72 h; *, *P* < 0.05 versus cells cultured for 24 h group. (**D**) NAD^+^/NADH ratio of C2C12 myoblasts (2×10^5^ cells/well), which were cultured for 24 h, and then treated by lactate (10 mM), pH6 medium, and 48 h medium (collected from C2C12 myoblasts which were cultured at 4×10^5^ cells/well for 48 h) for 24 h, respectively. *, *P* < 0.05 versus control group (Con, receiving no treatment). All data shown as mean ± SEM. Statistical tests were done by the Student's *t* test.

The concentrations of NAD^+^ and NADH and the size of the total NAD (NAD^+^ + NADH) pool in C2C12 myoblasts increased over time in culture (72 h compared to 24 h) (Figure 1A). Comparison of 72 h cultures at two different seeding densities showed that NAD^+^, NADH, and total NAD were also increased with cell density (Figure 1A). In addition, NAD^+^/NADH ratio of C2C12 myoblasts decreased with the extension of culture time or high seeding density (Figure 1B, 1C). A decrease in NAD^+^/NADH ratio was recapitulated by culturing C2C12 myoblasts under low pH, high lactate, or in conditioned medium from cells plated at high density for 48 h (Figure 1D). Thus, lactic acid buildup in the medium likely contributed to the redox shift observed over time in cell cultures.

### Rapamycin restored NAD^+^/NADH ratio in long-term cultured C2C12 myoblasts

**Figure 2:**
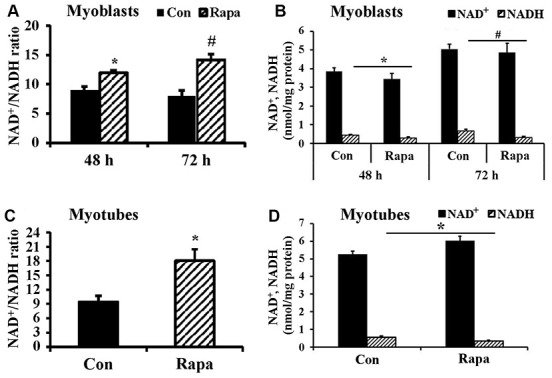
**Effect of rapamycin on NAD^+^/NADH ratio of longer-term cultured C2C12 myoblasts and myotubes.** (**A**) NAD^+^/NADH ratio of C2C12 myoblasts (2×10^5^ cells/well) cultured for 48 h and 72 h, then were treated by rapamycin (100 nM) for 24 h, respectively. *, *P* < 0.05 comparing control (cultured for 48 h followed by 24 h vehicle treatment) versus cells treated by rapamycin for 24 h group by Student’s *t* test. # *P* < 0.05 comparing control (cultured for 72 h followed by 24 h vehicle treatment) versus cells treated by rapamycin for 24 h group. (**B**) NAD^+^ and NADH concentration of C2C12 myoblasts. *, *P* < 0.05 control versus cells cultured for 48 h and then treated by rapamycin. #, *P* < 0.05 control versus cells cultured for 72 h and then treated by rapamycin. (**C**) NAD^+^/NADH ratio of C2C12 myotubes treated by rapamycin (100 nM) for 24 h. *, *P* < 0.05 control versus rapamycin-treated groups. (**D**) NAD^+^ and NADH concentrations of C2C12 myotubes treated by rapamycin (100 nM) for 24 h. *, *P* < 0.05 versus rapamycin-treated groups.

We cultured C2C12 myoblasts for 24-72 h and then subjected them to 24 h rapamycin treatment. Although the NAD^+^/NADH ratio of freshly plated C2C12 myoblasts (2×10^5^ cells/well, cultured for 24 h) was not significantly changed by 24 h rapamycin treatment (data not shown), rapamycin significantly increased the NAD^+^/NADH ratio and decreased NADH concentration of C2C12 myoblasts cultured for either 48 h or 72 h (Figure 2A, 2B). Similarly, rapamycin significantly increased the NAD^+^/NADH ratio (Figure 2C) and decreased NADH concentration (Figure 2D) in C2C12 myoblasts that had been cultured longer and differentiated into myotubes (*P* < 0.05). Therefore, rapamycin significantly affected NADH and NAD^+^/NADH ratio but not NAD^+^ (despite an uptrend in the differentiated myotubes) in C2C12 myoblasts cultured longer than 24 h.

### Effect of rapamycin on ATP concentration in C2C12 myoblasts and myotubes

**Figure 3:**
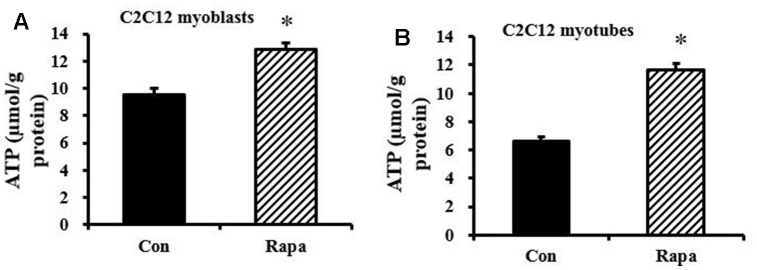
**Effect of rapamycin on ATP concentration in C2C12 myoblasts and myotubes.** (**A**) ATP concentration of C2C12 myoblasts, which were cultured for 48 h, then treated by rapamycin (100 nM) for 24 h. (**B**) ATP concentration of C2C12 myotubes, which were cultured for 6 d, then treated by rapamycin (100 nM) for 24 h. *, *P* < 0.05 control versus rapamycin-treated groups by Student’s *t* test.

Since decreased lactic acid production could indicate decreased glycolysis, and we and others previously showed that rapamycin can decrease mitochondrial respiration [30], these results suggested the possibility that rapamycin might be creating an energy deficit. However, rapamycin also inhibits many energy-consuming processes, making the net effect on energy balance unclear. We observed a significant increase in ATP concentration in rapamycin-treated C2C12 myoblasts (Figure 3A) and C2C12 myotubes (Figure 3B) (*P* < 0.05). Thus, rapamycin has a net ATP-sparing effect, despite reducing flux through pathways of energy production.

### Rapamycin did not change NAD^+^/NADH ratio and ATP concentration in kidney, liver, and muscle tissues of young mice

**Figure 4:**
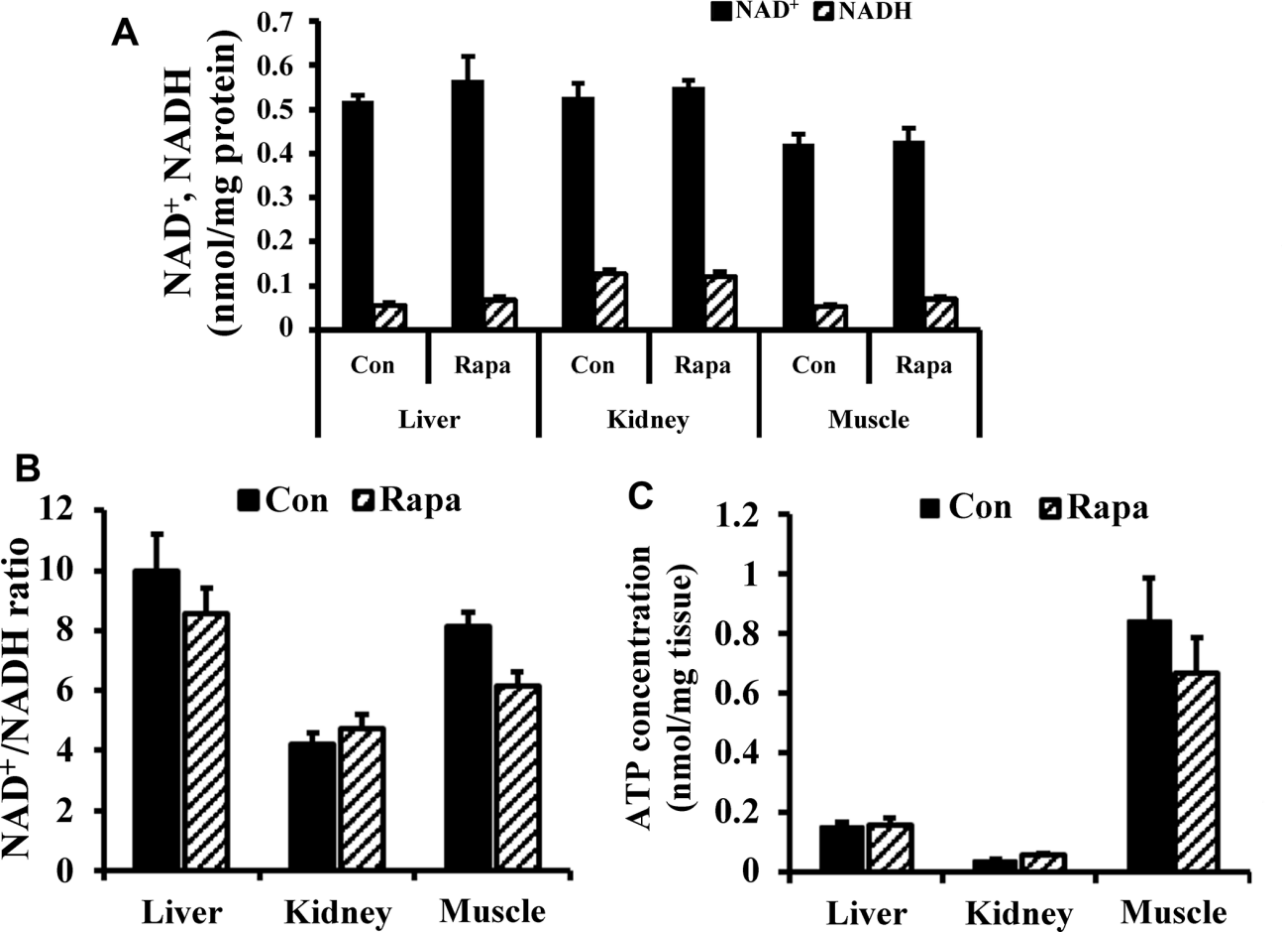
**NAD redox status and bioenergetics in kidney, liver, and muscle tissues of young mice.** (**A**) NAD^+^ and NADH concentrations, (**B**) NAD^+^/NADH ratio, and (**C**) ATP concentration in liver, kidney, and muscle tissues of young mice.

As shown in Figure 4A, 4B, rapamycin treatment did not significantly change the concentrations of NAD^+^ and NADH, or the NAD^+^/NADH ratio in kidney, liver, and muscle of young mice (2 months old). In addition, rapamycin treatment did not result in significant differences in ATP content in kidney, muscle, and liver (Figure 4C).

### Rapamycin induced a more oxidized state in old mouse muscle

We also employed optical redox imaging techniques to detect rapamycin’s effects on the NAD^+^/NADH redox status. Optical redox imaging measures the endogenous fluorescence intensities of NADH and Fp, which represents oxidized flavoproteins containing flavin adenine dinucleotide [26–29]. The optical redox ratio Fp/(NADH + Fp) reflects the mitochondrial redox state, and there is a linear correlation between optical redox ratio Fp/(NADH + Fp) and biochemically-determined redox ratio NAD^+^/(NADH + NAD^+^) [31, 32]. Thus, the optical redox ratio can be used as a surrogate indicator of NAD^+^/NADH redox state. Optical redox imaging has been widely applied to metabolic studies at both the cellular and tissue level [33–35]. Compared to extraction and biochemical determination of NAD^+^ and NADH, optical redox imaging directly shows NADH concentration and its spatial distribution of a tissue specimen with high resolution and can be used even when tissue is very limited.

**Figure 5:**
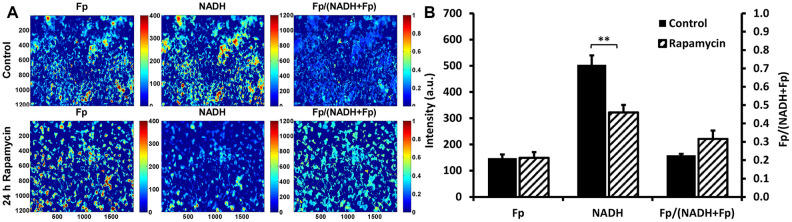
**Optical redox imaging of live C2C12 myoblasts.** (**A**) Typical redox images of control or rapamycin-treated cells, where the color bars in Fp or NADH images represent the intensities of the signals in arbitary unit and that for Fp/(NADH+Fp) represents the redox ratio ranging from 0 to 1; (**B**) Quantification of the redox imaging results (unpaired 2-tailed Student’s t test assuming unequal variance), n = 4. **, *P* < 0.01. All data shown as mean ± SEM.

Figure 5 shows that optical redox imaging can be used to detect the redox shift induced by 24 h 100 nM rapamycin in cultured undifferentiated live C2C12 myoblasts. Figure 5A displays typical Fp, NADH, and optical redox ratio images of control and rapamycin-treated C2C12 myoblasts, showing unchanged Fp signals (indicated by similar colors), decreased NADH (indicated by dark blue color for the majority of the cells) and increased optical redox ratio on average. Quantitative analysis (n = 4) revealed that 24 h 100 nM rapamycin treatment did not significantly change Fp level, but lowered NADH level in C2C12 by 36% (*P* < 0.01), resulting in an uptrend of the optical redox ratio Fp/(NADH+Fp) (*P* = 0.09) (Figure 5B). These results are consistent with rapamycin effects on C2C12 myoblasts obtained with biochemical analysis of NADH and the redox ratio (Figure 2).

**Figure 6:**
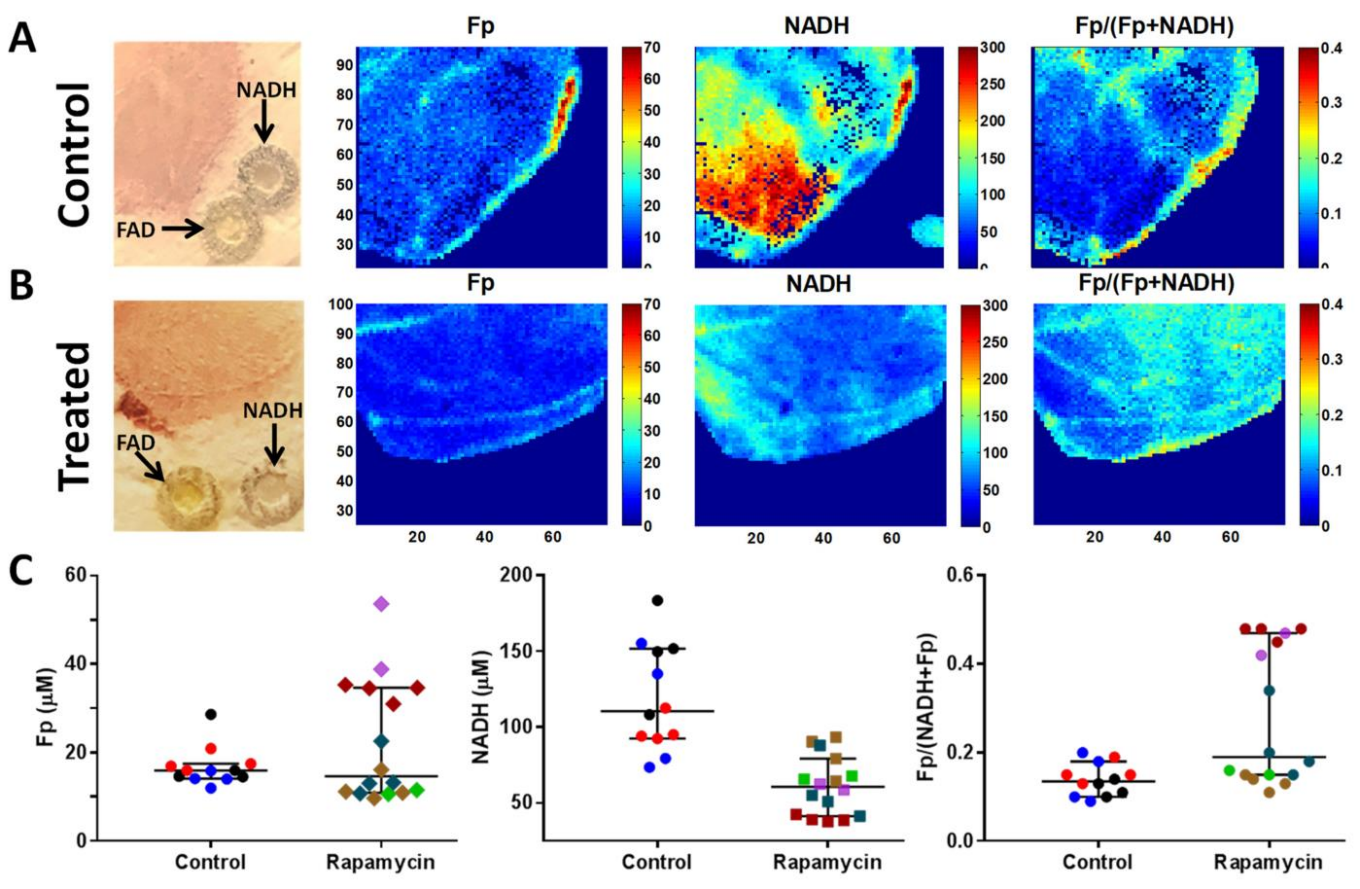
**Optical redox imaging of old mouse muscles.** (**A**, **B**) Representative white light and redox images of one layer of a quadriceps specimen from control (**A**) and treated (**B**) groups. The in-plane spatial resolution of the redox images is 100 μm. The concentrations of Fp and NADH are nominal concentrations in reference to the embedded flavin adenine dinucleotide (FAD) and NADH standards, respectively. The color bars for Fp and NADH images are nominal concentrations in reference to the embedded standards, respectively, and that for the redox ratio image ranges from 0-0.4. (**C**) Scattered plots of redox indices (Fp, NADH, and the redox ratio) for all specimens comparing control and treated groups. Each dot is a mean value for a specific redox index of a tissue layer and the tissue layers from the same tissue specimen are encoded with the same color (bars: median with 95% CI).

Next, we employed multi-section optical redox imaging *ex vivo* to interrogate the redox states of snap-frozen muscles from aged mice (17 months). Figure 6 depicts representative redox images of one section (layer) of a quadriceps specimen from control (Figure 6A) and treated (Figure 6B) groups and the scatter plots for all specimens (Figure 6C). Quantitative analysis using a linear mixed model for statistical comparison between these two groups (Table 1) showed that rapamycin treatment lowered NADH level significantly (*P* < 0.01) and resulted in a more oxidized state (larger redox ratio) with a marginal *P* value of 0.051 in quadriceps.

**Table 1:** Estimated mean ± SEM of the redox indices in quadriceps muscles.

**Group**	**Fp (μM)**	**NADH (μM)**	**Fp/(NADH+Fp)**
Control	25 ± 5.4	141 ± 10	0.091 ± 0.073
Rapamycin	18 ± 5.0	47 ± 9.8	0.31 ± 0.059
*P*	0.15	< 0.01	0.051

## DISCUSSION

Redox balance is necessary for optimum cellular health across the lifespan. The intracellular NAD^+^/NADH redox state reflects the metabolic balance of the cell in generating ATP through glycolysis and oxidative phosphorylation in mitochondria [36]. Importantly, it has now been clearly demonstrated that cellular NAD^+^ levels decline during aging [19]. NAD^+^ and NAD^+^/NADH decrease and NADH increases with age in human brains as measured by high-field MRS *in vivo* [24]. Similarly, a shift toward a more reduced NAD^+^/NADH ratio and a decline in NAD^+^ have been reported across multiple tissues in aged rats [22]. These changes may play a crucial role in the development of metabolic dysfunction and age-related diseases [37–39]. Zhang et al. reported that NAD^+^ repletion enhanced life span in mice [8], and restoring NAD^+^ has benefits in cultured cells [40] and aging mammalian tissues [41]. Calorie restriction, which extends healthy lifespan, has been reported to increase tissue NAD^+^ concentrations [42, 43] and has also been suggested to work in part by raising the intracellular NAD^+^/NADH ratio [36]. These observations support the model that a higher NAD^+^/NADH ratio and lower NADH level maintained by rapamycin might contribute to a more youthful metabolic state. Results from the present study provide initial evidence for a beneficial effect of rapamycin-treatment on NAD^+^/NADH redox balance, i.e., lower NADH levels in long-term cultured C2C12 mouse myoblasts *in vitro* and quadriceps of old mice *ex vivo*. More research is needed to further verify these findings and elucidate the mechanism by which rapamycin reverses NAD^+^/NADH imbalance and decreases NADH levels *in vivo*.

The NAD^+^/NADH redox state within a single cell is influenced by the metabolic state of that cell as established by the flux of redox-active metabolites, such as lactate, pyruvate, and ketone bodies [44]. Lactate production is closely linked to the pH of cell medium and NAD^+^/NADH redox state, and lactate-mediated signaling is influenced by the NAD^+^/NADH redox state [45]. Increasing level of L-lactate suppressed the proliferation of murine and human T cells [46]. Patients suffering from mitochondrial disease can exhibit a sensitivity to lactic acidosis. Aging cells exhibit some degree of mitochondrial dysfunction [47, 48], and senescent cells accumulate more lactate than young cells [23]. In the present study, we observed that lactate treatment decreased the NAD^+^/NADH redox ratio and rapamycin decreased NADH and maintained the NAD^+^/NADH redox state of long-term cultured C2C12 myoblasts *in vitro* and the quadriceps of old mice. It has been shown that rapamycin suppresses both glycolysis and geroconversion, and decreases lactate production independent of respiration in proliferating cells and senescent cells and in the presence of the oxidative phosphorylation inhibitor oligomycin [23]. Thus, rapamycin may maintain NAD^+^/NADH redox balance of cells in part via decreasing lactate production [49, 50].

ATP is a direct cellular energy source and is responsible for a wide variety of cellular activities, including cell proliferation, metabolism, and survival. Mitochondrial NADH drives ATP synthesis by donating electrons to complex I of the electron transport chain. Rapamycin is known to decrease mitochondrial respiration [51, 52]. Thus, a more oxidized redox state in the presence of rapamycin might reflect decreased capacity for mitochondrial ATP synthesis, which together with a decrease in glycolytic ATP production might lead to energetic stress. Interestingly, in the present study, we found significant increases in both NAD^+^/NADH ratio and ATP content in long-term cultured C2C12 myoblasts and myotubes after rapamycin treatment. This suggests that energetic demand is decreased in the presence of rapamycin, rather than the capacity for ATP synthesis. Consistently, Ye et al. reported that the doses of rapamycin required to extend life do not cause obvious mitochondrial dysfunction in skeletal muscle of mice [53]. Furthermore, low-dose rapamycin extends lifespan in a mouse model of mitochondrial genome depletion syndrome [54]. Collectively, these results suggested that rapamycin does not impair bioenergetics or mitochondrial function, but instead reduces flux through energy-consuming pathways.

In summary, this study suggests that rapamycin favors a more oxidized NAD^+^/NADH ratio and improves ATP availability, most likely by reducing its demand for biosynthetic processes. Further studies will be needed to elucidate the underlying mechanisms. This study provides new insight into the potential mechanisms by which rapamycin might influence the aging process, and suggests that redox imbalances may be an indication that can be targeted using rapamycin to improve health and longevity among the aging population.

## MATERIALS AND METHODS

### Reagents

Rapamycin was purchased from Calbiochem (Billerica, MA, USA, Cat. No. 553210). Dulbecco's modified Eagle's medium (DMEM), fetal bovine serum (FBS), and horse serum were obtained from Invitrogen (Grand Island, NY, USA). ATP Determination Kits were purchased from Invitrogen (Cat. No. A22066). Micro BCA Protein Assay Kits were purchased from Thermo Fisher Scientific (Cat. No. 23235). Other chemicals were purchased from Sigma (St. Louis, MO, USA) unless noted.

### Animal and treatment

All experiments were approved by the Institutional Animal Care and Use Committee at the University of Pennsylvania. Male C57BL/6 mice were obtained from Taconic at approximately 8 weeks or 17 months of age. Mice were kept in a specific pathogen-free barrier facility with controlled temperature and humidity and a 12-h/12-h light/dark cycle. Mice were fed standard chow diet.

Acute rapamycin treatments were performed by injecting mice (8 weeks old) intraperitoneally once daily with rapamycin (2 mg/kg) or vehicle (saline) for 2 days. The dose was selected to approximate the amount of rapamycin that has been shown to extend life when delivered in the diet [6] and is the same dose that we have employed in prior studies to demonstrate inhibition of mTOR complexes and induction of glucose intolerance *in vivo* [45]. At 1 h after the second injection the mice were sacrificed and liver, kidney, and muscle tissues were collected.

Seventeen months old mice were treated with rapamycin (2 mg/kg) intraperitoneally twice. The first injection was 24 h before sacrifice and the second injection was 1 h before sacrifice. Quadriceps was removed within several minutes after sacrificing the animal and snap-frozen immediately with liquid nitrogen for optical redox imaging analysis.

### C2C12 myoblast culture

The C2C12 mouse myoblasts (ATCC, CRL-1772) were grown in DMEM supplemented with 4.5 g/L D-glucose, 2 mM glutamine, 10% FBS and 1% penicillin/streptomycin (Thermo Scientific). Cells were maintained at 37 °C in a 5% CO_2_ humidified incubator. The cells were treated for 24 h with 100 nM rapamycin, which has previously been shown to inhibit respiration [13].

### C2C12 myotube culture

The C2C12 mouse myoblasts were cultured with DMEM supplemented with 4.5 g/L D-glucose, 2 mM glutamine, 10% FBS and 1% penicillin/streptomycin (Thermo Scientific) in 6 wells plates (2×10^5^ cells/well). When the cell confluence reached 90%, the cells were washed once with PBS and the media was replaced with DMEM containing 2% horse serum (Invitrogen) and 1 μM insulin (Novo-Nordisk) to induce differentiation into myotubes. The medium was replaced every 24 h for 5 days, whereupon the mature myotubes were moved to maintenance media (2% horse serum without supplemental insulin) and treated with vehicle or rapamycin (100 nM) for 24 h.

### NAD^+^ and NADH metabolite extraction from myoblasts and myotubes

After aspirating the media from the culture plate (24 wells), 200 μL 0.6 M perchloric acid was added (for NAD^+^ content assay) or 0.25 M KOH and 50% ethanol mixture was (for NADH content assay), and the extracts were collected in 0.5 mL tubes. KOH extracts were heated at 55 °C for 10 min, and all tubes were centrifuged at 15,000 g for 10 min at 4 °C. The clear supernatant was diluted to 1:10 in ice-cold 100 mM phosphate buffer, pH 8 [55]. Precipitate pellets in the perchloric acid tubes were collected for protein concentration quantification [54].

### NAD^+^ and NADH metabolite extraction from tissues

NAD was extracted from 50 mg of clamp-frozen kidney, liver, and muscle tissues in 0.6 M perchloric acid at 4 °C using a tissue lyzer (Qiagen) with a metal bead set at 20 Hz for 2 min. The insoluble materials were pelleted by centrifugation at 16000 g for 10 min at 4 °C for protein assay by the BCA method. The supernatant was diluted to 1:100 in ice-cold 100 mM phosphate buffer (pH 8.0) for NAD assay [55].

NADH was respectively extracted from 50 mg of clamp-frozen kidney, liver, and muscle tissues in ice-cold extraction buffer (25 mM NH_4_Ac, 25 mM NaOH, 50% (v/v) acetonitrile) flushed with nitrogen gas. Lysates were mixed 1:1 (v/v) with ethanol-KOH extraction buffer (250 mM KOH, 50% (v/v) ethanol-KOH), then heated at 55 °C for 10 min. The supernatants were diluted 1:50 in prechilled 100 mM phosphate buffer (pH 8.0) for NADH assay [55].

### NAD^+^ and NADH concentration measurement

NAD^+^ and NADH were immediately measured after extraction by an enzymatic cycling assay in a 96-well format as described previously with modifications [44]. Briefly, 5 μL of NAD^+^ standards, diluted cell extracts were combined respectively with 95 μL of cycling mixture (2% ethanol, 100 μg/mL alcohol dehydrogenase, 10 μg/mL diaphorase, 20 μM resazurin, 10 μM flavin mononucleotide, 10 mM nicotinamide, 0.1% bovine serum albumin in 100 mM phosphate buffer, pH 8.0). The cycling reaction was carried out at room temperature for 30 min, and resorufin accumulation was measured by fluorescence excitation at 544 nm and emission at 590 nm [56]. The measured NAD and NADH concentrations were normalized according to protein concentrations. C2C12 myoblast and C2C12 myotubes protein content were quantified using the Micro BCA Protein Assay Kit (Thermo Scientific).

### ATP assay

The concentrations of ATP in the cultured cells and tissues were assayed using Molecular Probes ATP Determination Kit (Invitrogen, A22066) according to the manufacturer’s protocol.

### Optical redox imaging of quadriceps

Quadriceps tissue embedding and redox scanning were conducted as previously reported [57]. Briefly, frozen quadriceps was embedded in mounting buffer (freezing point -30 °C) adjacently to FAD and NADH standards (100 μM each). The embedded tissues were shaved flat and raster scanned under liquid N_2_ with the Chance redox scanner to obtain fluorescence signals from NADH and Fp (oxidized flavoproteins containing FAD) [29, 58]. Two to four layers spacing 200 μm were scanned for each tissue specimen. Five of rapamycin-treated quadriceps and three specimens of control were scanned with an in-plane spatial resolution of 100 μm.

The data generated by optical redox scanning of quadriceps were first processed with a customized Matlab^®^ program. For each layer, the intensities of NADH and Fp signals were first thresholded at the signal-to-noise ratio of 3 above the image background, and then nominalized to the internal frozen standards to obtain the images of nominal concentrations. The image of redox ratio Fp/(NADH+Fp) was then generated pixel-by-pixel from these two nominal concentration images. The mean values and the standard deviations of all the redox indices (Fp, NADH, Fp/(NADH + Fp)) were then obtained for each layer.

### Optical redox imaging of C2C12 myoblast treated with rapamycin

Glass bottom dishes (Cellvis, D35-20-1.5-N) were used to culture the C2C12 myoblast cells for optical redox imaging *in vitro*. To avoid the high fluorescence background of the regular culture medium, forty five minutes before imaging, C2C12 myoblasts were rinsed with PBS twice followed by adding 1 mL of Live Cell Imaging Solution (LCIS, Invitrogen™) spiked with glucose (final concentration 22 mM) and glutamine (final concentration 2 mM). The dishes were imaged alternately between the control and rapamycin-treated groups using a fluorescence microscope (Axio Observer.Z1/7, Zeiss). The objective lens was 20× (0.8 NA). For NADH channel, the optical filters were 370-400 nm and 414-450 nm for excitation and emission, respectively. For Fp channel, the optical filters were 450-488 nm and 500-530 nm for excitation and emission, respectively. To confirm the collected signals reflect the redox state, redox response was obtained by first imaging the baseline of the cells, followed by sequential addition of a) carbonilcyanide p-triflouromethoxyphenylhydrazone (FCCP, 0.5 μM final concentration) and b) rotenone (ROT, 1 μM final concentration) together with antimycin A (AA, 1.25 μg/ml final concentration) and imaging accordingly, where treatment of either a) or b) was ~5 min [59]. It is known that FCCP as a mitochondrial oxidative phosphorylation uncoupler causes oxidation of NADH, resulting in a decrease in NADH. ROT and AA inhibits complex I and complex III, respectively, preventing NADH from being oxidized, thus resulting in an increase in NADH. By sequential addition of mitochondrial uncoupler FCCP and inhibitors ROT+AA and imaging accordingly, we observed expected responses of C2C12 myoblasts (data not shown), confirming that C2C12 myoblasts under the experimental settings were sensitive to redox modulations and the detected NADH and Fp signals reflected the redox state.

The data acquired from optical redox imaging of C2C12 cultures were processed with Matlab^®^ as previously described [59, 60]. Each image was first flattened with a surface fit function (poly33), followed by background subtraction and thresholding. For background subtraction, a cell-free area was chosen. For thresholding, signals with value smaller than 3 times of the standard deviation of the background were excluded. The mean value for each field of view (FOV) was calculated for all the redox indices (Fp, NADH, Fp/(NADH+Fp)) and then averaged across all FOVs (3-5) of each dish.

### Statistical analysis

Results are shown as mean ± SEM (standard error of mean). Data were analyzed by unpaired 2-tailed Student’s t test. For the redox scanning data of quadriceps tissues, statistical analysis was performed with a linear mixed model using SAS 9.4 (SAS Institute, Cary, NC, USA), where tissue ID was set as subject, layers as repeated measure, unstructured as covariance structure, and treatment as fixed effect. Significance was set at *P* < 0.05.
